# A Reliable BioFET Immunosensor for Detection of p53 Tumour Suppressor in Physiological-Like Environment

**DOI:** 10.3390/s20216364

**Published:** 2020-11-08

**Authors:** Chiara Baldacchini, Antonino Francesco Montanarella, Luca Francioso, Maria Assunta Signore, Salvatore Cannistraro, Anna Rita Bizzarri

**Affiliations:** 1 Biophysics and Nanoscience Centre, DEB, Università degli Studi della Tuscia, 01100 Viterbo, Italy; baldacchini@unitus.it (C.B.); antonino.m@unitus.it (A.F.M.); bizzarri@unitus.it (A.R.B.); 2 CNR-IMM Institute for Microelectronics and Microsystems, Via Monteroni, University Campus, Bld. A/3, 73100 Lecce, Italy; lucanunzio.francioso@cnr.it (L.F.); mariaassunta.signore@cnr.it (M.A.S.)

**Keywords:** p53, EGFET, immunosensors, BioFET

## Abstract

The concentration of wild-type tumour suppressor p53_wt_ in cells and blood has a clinical significance for early diagnosis of some types of cancer. We developed a disposable, label-free, field-effect transistor-based immunosensor (BioFET), able to detect p53_wt_ in physiological buffer solutions, over a wide concentration range. Microfabricated, high-purity gold electrodes were used as single-use extended gates (EG), which avoid direct interaction between the transistor gate and the biological solution. Debye screening, which normally hampers target charge effect on the FET gate potential and, consequently, on the registered FET drain-source current, at physiological ionic strength, was overcome by incorporating a biomolecule-permeable polymer layer on the EG electrode surface. Determination of an unknown p53_wt_ concentration was obtained by calibrating the variation of the FET threshold voltage versus the target molecule concentration in buffer solution, with a sensitivity of 1.5 ± 0.2 mV/decade. The BioFET specificity was assessed by control experiments with proteins that may unspecifically bind at the EG surface, while 100pM p53_wt_ concentration was established as limit of detection. This work paves the way for fast and highly sensitive tools for p53_wt_ detection in physiological fluids, which deserve much interest in early cancer diagnosis and prognosis.

## 1. Introduction

The transcription factor p53 is considered the most important tumour suppressor for its pivotal role in many cellular processes inducing a number of genes implicated in DNA repair, cell-cycle arrest and apoptosis [1,2]. More than half of human cancers harbour p53 mutations, this reducing wild-type p53 (p53_wt_) concentration in cells and extracellular physiological fluids in patients with cancer, with respect to the standard nanomolar range characterizing healthy tissues [3,4]. On the other side, high concentrations of p53_wt_ protein in blood have been correlated to asbestosis, cancer of lungs and mesothelioma, even many years before the malignancy became clinically overt [5,6,7]. Thus, p53_wt_ concentration in cells and blood deserves a clinical significance for the early diagnosis of many important diseases. As a consequence, a variety of techniques have been proposed for p53_wt_ protein assay, including, but not limited to, colorimetry, chemiluminescence, electrochemiluminescence, immunochromatography, enzyme-linked immunosorbent assays (ELISA), surface-enhanced Raman spectroscopy (SERS), surface plasmon resonance (SPR), electrochemistry, standard and field-effect transistor (FET) based amperometry [3,4,8,9,10,11,12,13,14,15].

In the last years, among various analytical methods, biosensing based on FET technology (also known as BioFET) has drawn the attention of the scientific community for its outstanding properties, both on the basis of sensing performance (i.e., sensitivity, specificity and fast response to target concentration) and of the effective cost and possibility of mass production thanks to semiconductor technology [16,17]. Most importantly, BioFET sensors would overcome issues related to target structural modifications by label introduction, which, in turn, could affect the biosensor recognition ability. Finally, BioFET does not need any optical devices for readout, with significant reduction of the biosensor dimensions.

In a FET device, the current flow between two suitably polarized terminals (drain and source electrodes), embedded in a semiconductor substrate, is finely modulated by the potential of a third element, the gate electrode. In BioFET, the gate electrode is in contact with an electrolyte solution in which a stable reference electrode is placed to set the gate surface potential constant. The gate surface is suitably biofunctionalized with receptor molecules that can specifically recognize and capture the target molecules in the solution. If the target molecules are endowed with a net charge, which depends on both their isoelectric point (pI) and the solution pH, capturing of the charged targets onto the FET gate surface results into a variation of its potential due to the charge variation that, in turn, affects the measurable drain-source current [18]. Therefore, a correlation between such a current and the target concentration would allow an unknown biomarker concentration in solution to be determined.

Although huge efforts have been devoted to the implementation of commercial BioFET, two main drawbacks have limited, up to date, the development of low-cost and disposable devices. The first originates from the presence of ion species in physiological solutions that hinder the target charge to affect the gate surface potential, above the so-called Debye screening length. This length characterizes the charge screening in electrolytes under the Debye–Hückel model, and mainly depends on the working solution temperature and ionic strength [17,19,20]. In physiological solutions and ambient temperatures, the Debye screening length is in the order of one nanometer. Since the biomolecules involved in biorecognition processes at the basis of target molecule capture may largely exceed this length, a drastic electrostatic screening of the target charges may occur, with minimal or no effect on the gate surface potential [21]. Buffer dilution reduces the ionic strength of the solution and extends the Debye length; however, this can cause severe issues such as instability of the biomolecules in solution, or reduced affinity of the capturing–target molecule interaction [17]. Nevertheless, it is worth noting that, in some cases, biosensing by means of BioFET has been reported well beyond the Debye length, in high ionic strength solutions, and this has been attributed to the formation of a Donnan equilibrium within the adsorbed target layer, which modifies the charge distribution at the interface, affecting either the interface capacitance [22] or the surface potential [19]. However, a series of loopholes emerged to overcome the Debye screening adverse effect in physiological solutions, which were based on the use of short receptors, such as aptamers [23], or nanostructured gate electrodes, such as silicon nanowires or carbon nanotubes [24,25]. Also, a particular sensing layer based on long chain of polyethylene glycol (PEG) polymer has been successfully implemented to extend the Debye length [26,27,28], thanks to a newly induced Donnan equilibrium [29].

A second drawback is related to the coupling difficulties arising when a semiconducting electronic device (FET) is to be employed in a wet environment. Accordingly, a new device design (EGFET) has been proposed, in which the FET gate is extended through a suitable conductive electrode, the so-called extended gate (EG) [28,30,31]. In such a configuration, a suitably microfabricated electrode, whose upper part can be functionalized with target-capturing molecules, is exposed to the target-containing solution, while the other side of this electrode is placed in contact with the internal FET gate terminal. In such a way, the charge variation effect occurring upon biorecognition is transmitted to the FET gate, resulting analogously in the I_ds_ variation. Then, contamination of the FET silicon die is avoided and the FET can be reused, while the disposable EG can be removed after the measurement.

In this paper, we present a label-free EGFET immunosensor for the detection of p53_wt_, which benefits from both the extended-gate configuration, in terms of disposability and low-cost production, and the introduction of a suitable PEG layer for the enhancement of the device sensitivity as due to an extended Debye length. Such a BioFET design allowed us to reach a limit of detection (LOD) of 100 pM for p53_wt_ in physiological solutions at room temperature. The BioFET detection range spans over three orders of magnitude (0.1–10 nM), with a sensitivity of 1.5 ± 0.2 mV/decade. All these features make the proposed biosensor particularly suitable for the assays of p53_wt_ at the concentration range expected in cancer cells with p53 gene mutation (0.1 nM), as well at concentrations expected in healthy cells (1 nM), and beyond.

## 2. Materials and Methods

### 2.1. Experimental Setup

**Figure 1:**
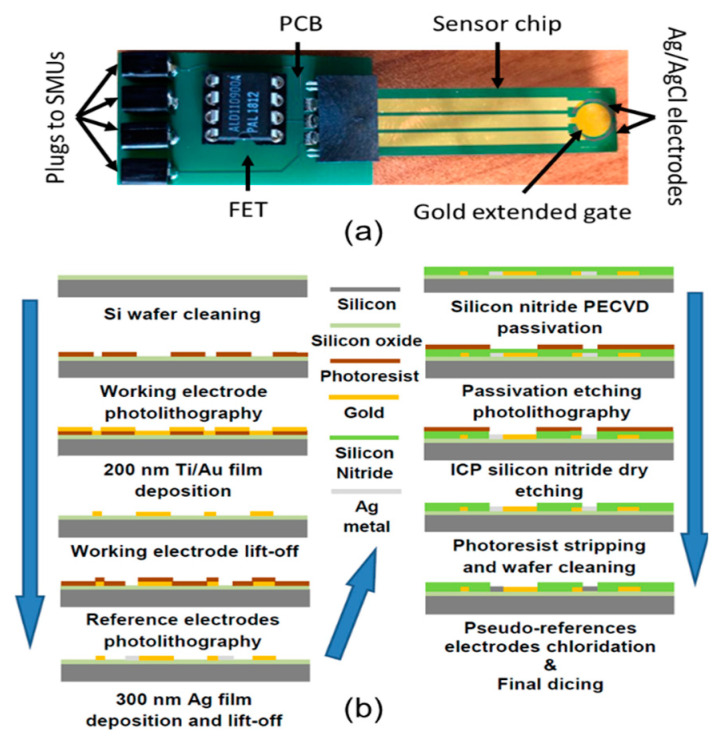
(**a**) The sensor chip (10 × 40 mm^2^ outer dimensions), with the extended-gate gold electrode (sensing area 20 mm^2^) and two Ag/AgCl pseudoreferences (dark semicircular area around the gold sensing area), connected to the home-made printed circuit board, where the commercial n-type MOSFET ALD110900A is plugged in and four plugs are available to connect the printed circuit board (PCB) with the source-meter units (SMUs). (**b**) Sketch of the microfabrication process of the sensor chip.

To reduce the FET-to-EG distance, contact resistances and ambient noise, a commercial zero-threshold n-type metal oxide semiconductor field-effect transistor (MOSFET; ALD110900A from Advanced Linear Devices Inc., Sunnyvale, CA, USA) was integrated in a printed circuit board (PCB) with a socket for connecting the sensor chip (which contains the gold EG and two pseudoreference electrodes, see next Section 2.2) and plugs for wiring the probe station (Figure 1a). The sensor chip was vertically immersed in a custom fluid cell, together with a commercial bulky Ag/AgCl reference electrode (DriRef-2, World Precision Instruments Ltd, Hitchin, UK). The fluid cell had the dimensions of 1.0 × 0.5 × 3.5 cm^3^, and it was fitted into a heavy plastic support during measurements, to ensure stability and avoid mechanical noise. At a height of 1.5 cm from the bottom of the fluid cell, a lateral hole allowed the injection of the target solutions into the cell during measurements. The maximum capacity of the cuvette was 600 μL. The experimental measurements were carried out by a Keithley 2636B (Tektronix, Beaverton, OR, USA), which have a sensitivity in the fA range for current measurements and μV range for potential measurements. Two source-meter units (SMUs) of the Keithley apparatus were used. The SMU1 applied the voltage between the drain and the source electrodes (V_ds_), which was kept constant at 100 mV for all the experiments, and measured the corresponding current flow (I_ds_). The SMU2 applied a potential bias (V_ref_) to the reference electrode in solution.

### 2.2. Sensing Chip Microfabrication

The sensing chip was microfabricated through the procedure shown in Figure 1b, in order to have the gold EG and two Ag/AgCl pseudoreference electrodes on the same silicon chip. The manufacturing procedure was carried on thermal oxidized (100) 4” silicon wafers (n-type) accurately cleaned with an acetone and 2-propanol ultrasonic bath for 15 min and then rinsed in deionised water, followed by hotplate dehydration at 120 °C for 600 s. A single layer of AZ5214 reversal image (negative tone) photoresist (MicroChemicals GmbH, Ulm, Germany) about 1.4 µm-thick was dispensed on the silicon substrate and soft-baked at 110 °C for 50 s. The patterning of the photoresist was realized by 365 nm optical lithography process in hard contact mode with a Karl Suss MA6 (Suss Microtec, Waterbury Center, VT, USA) tool. The photoresist layer was exposed with a 5” quartz mask with chromium patterns for the realization of the working electrode; after reversal image procedure completion (for AZ5214), the sample was dipped into AZ326 developer to remove soluble photoresist areas. In order to completely remove photoresist residuals from silicon exposed surface and guarantee highly reproducible subsequent etching process, a final five-second spray step with AZ326 developer was added to standard procedure. After photolithographic steps, a Cr/Au thin film (5 nm/200 nm) was deposited by e-beam evaporation, followed by lift-off to pattern the working electrode and the contact paths for reference electrodes (see process graphic Figure 1). A second lithography step with the same recipe was performed for the deposition of the silver reference electrodes, completed by a 300 nm silver deposition by e-beam evaporator. After the fabrication of all electrodes, the wafer was fully passivated by 400 nm of ultra-low residual stress (+50 MPa) PECVD silicon nitride, deposited at 350 °C. A final photolithography process allows to selectively etch the silicon nitride layer by Inductively Coupled Plasma process with a SF_6_/O_2_ mixture to expose working and reference electrodes to the solution. A final measurement of the gold working electrode before and after the plasma process, confirms the average final roughness of about 5 nm. Figure 1 also shows the fabricated electrode with two embedded pseudoreference electrodes around the circular working electrode. The electrochemical chloridation of the silver electrodes to Ag/AgCl was performed in 1% NaCl aqueous solution with an applied DC current density of 0.003 mA/mm^2^ for 90 s, followed by multiple rinses in deionized water. These Ag/AgCl microfabricated pseudoreference electrodes were introduced in the sensing chip design to improve the compactness of the experimental setup; this deserving interest in connection with the developing of future devices. However, preliminary, careful experiments conducted by using these electrodes to set the gate potential to a fixed value, evidenced a tension offset with respect to the bulky commercial electrode (see previous Section 2.1) of about 100 ± 20 mV. Therefore, in order to provide experimental results comparable with those reported in the literature with the same MOSFET working also in liquid environment [32], we decided to conduct experiments by using a bulky commercial electrode as reference electrode.

### 2.3. Extended-Gate Electrode Surface Biofunctionalization

**Figure 2:**
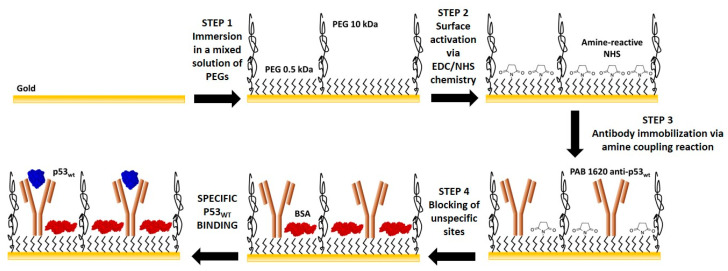
Main steps of gold extended-gate electrode biofunctionalization procedure (not to scale).

Prior to functionalization, the EG gold electrode was cleaned with H_2_O_2_ (Merck Millipore, Darmstadt, Germany) under ultraviolet (UV) light for 30 min, according to the so-called liquid-based hydrogen peroxide-mediated UV-photooxidation (liquid-UVPO) technique [33]). Then, it was rinsed with deionized water and dried with nitrogen. The biofunctionalization procedure (Figure 2) was performed essentially in agreement with that reported by Tarasov and coworkers [28]. It consists of four steps, each one separated by the following by rinsing the EG surface with phosphate-buffered saline (PBS) 1X (147 mM) solution at pH 7.4 for 15 min, to remove unbound molecules, successive rinsing with filtered (0.2 μm filtering membrane pore size) deionized water, and drying by pure nitrogen. First, the EG electrode was incubated with a mixed solution of SH-PEG-COOH (0.5 kDa; Biochempeg, Watertown, MA, USA), and SH-PEG (10 kDa; Merck Millipore), at a concentration ratio of 40 μM to 2 μM, respectively, in PBS 1X solution at pH 7.4, for one hour at room temperature. Furtherly, it was incubated with N-ethyl-N′-(3-(dimethylamino) propyl) carbodiimide (EDC) and N-hydroxysuccinimide (NHS), both purchased from GE Healthcare (Uppsala, Sweden), at 0.4 M/0.1 M ratio in PBS 1X solution at pH 7.4, for 20 min at room temperature. Then, the EG electrode was incubated overnight at 4 °C with the target-capturing p53 antibody (mouse monoclonal anti-p53wt, PAb1620 clone antibody; Merck Millipore) at a concentration of 13 nM in PBS 1X solution at pH 7.4. Finally, to avoid nonspecific binding, the unbound sites on the electrode surface were blocked by incubating it with a 1 mg/mL bovine serum albumin (BSA; Merck Millipore) solution in PBS 1X solution at pH 7.4 for one hour at room temperature.

### 2.4. Biosensing Experiments

All the biosensing experiments were performed by using PBS 100 mM solution at pH 8.0 as working buffer. The ionic strength of the working buffer was chosen to mimic the screening length of physiological solutions [26], while the pH value was selected somewhat higher than the physiological one (7.4), in order to increase the net charge of the p53_wt_ target molecules. Indeed, p53_wt_ (Genscript, Piscataway, NJ, USA) has a pI of 6.3 in its unphosphorilated state [15], and thus it will bear a negative net charge at pH 8.0 [17]. Due to the geometry of the EG sensing area (circular gold area on the top of the sensor chip in Figure 1a) and of the fluid cell, the starting buffer volume was set at 350 μL, to ensure the complete immersion of the EG electrode area. The electrolyte solution was not purged to remove dissolved oxygen and was exposed to the atmosphere during the measurements.

Each biosensing experiment consisted of three steps: (i) the BioFET transfer curve (I_ds_–V_ref_ curve) was acquired by measuring the MOSFET I_ds_ as a function of V_ref_, as applied by the reference electrode, while it is dipped in the electrolyte solution; (ii) I_ds_ at constant both V_ds_ (100 mV) and V_ref_ (400 mV) was consecutively measured, until a stable signal was reached; (iii) I_ds_ at constant V_ds_ (100 mV) and V_ref_ (400 mV) was consecutively measured while small volumes (20 μL) at increasing concentrations of p53_wt_ were added by pipetting into the fluid cell (the same working buffer was used, in order to avoid current responses as a result of change in pH or ionic strength in the electrolyte [34]).

Transfer curves from nine different experiments were used to calculate the average curves. Per each tested p53_wt_ concentration, five different biosensing experiments were carrier out. Data analysis, plotting and fitting were performed by using the Microcalc OriginPro 8.5 software product (OriginLab Corporation, Northampton, MA, USA).

## 3. Results and Discussion

### 3.1. BioFET Transfer Characteristics

**Figure 3:**
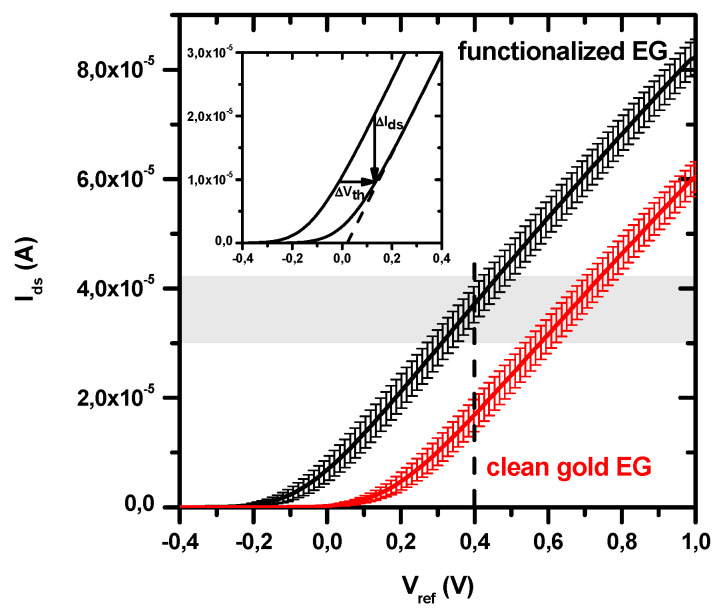
BioFET average transfer curves (MOSFET I_ds_ as a function of the V_ref_ applied by the reference electrode), with standard deviations, obtained by using as extended gate (EG) a biofunctionalized gold electrode (black curve) or a bare gold one (red curve). The V_ref_ applied during the biosensing experiments (400 mV) is highlighted by a vertical dashed line. The range of the starting I_ds_ values (around ten μA) is highlighted by the grey area. Inset: both extraction of the threshold voltage (V_th_) from a representative curve, by a linear extrapolation method [35], and the correspondence between I_ds_ and V_th_ changes are shown.

Figure 3 shows the average transfer curve I_ds_–V_ref_ of the BioFET as obtained when the EG electrode is functionalized with p53_wt_ specific antibody (black curve). For comparison, the average transfer curve obtained with bare gold EG surface is also shown (red curve). The slope in the linear regime of the single transfer curves, corresponding to the g_m_ transconductance [17]), has been obtained by a linear fit of the curves in the 300–500 mV range. The obtained average values are (70.5 ± 2.7) × 10^−6^ A/V when the EG electrode is clean, and (79.6 ± 1.2) × 10^−6^ A/V when it is biofunctionalized.

The threshold voltages (V_th_) of the transfer curves have been obtained by applying a linear extrapolation method [35], as shown in Figure 3 (inset). Their average values are (+160 ± 30) mV for clean gold EG electrode, and (−70 ± 40) mV for biofunctionalized gold EG electrode. These deviations from the zero-threshold which characterizes the commercial n-type MOSFET used, could be attributed to a combination of three possible effects: (i) the presence of the electrical double layer (EDL) which is formed in the electrolytic solution; (ii) the presence of the different molecules involved in the electrode functionalization; (iii) changes in the flat band voltage, somewhat correlated to the previous mechanisms [34,36]. In particular, the shift of the threshold (and obviously of the transfer curve) toward positive values, as observed for the clean gold electrode dipped in the electrolytic solution (see Figure 3), is indicative of the accumulation of negative charges at the EG electrode surface. Indeed, when an n-type MOSFET is used as transduction element, the accumulation of negative charges on the EG surface (and then on the FET gate) induces holes in the semiconductor channel and, thus, a decrease of I_ds_ measured at a fixed V_ref_. Conversely, the threshold shift toward negative values, as registered for the functionalized EG electrode, implies the accumulation of positive charges at this electrode.

Based on the obtained transfer curves, we set the working V_ref_ at 400mV, to ensure that the n-type MOSFET is working in the linear regime conditions. In this way, each variation of the recorded I_ds_ will be proportional to the corresponding shift in the V_th_ (see inset in Figure 3), with the transconductance g_m_ being the proportional constant (ΔI_ds_ = −g_m_ ΔV_th_)) [17].

### 3.2. Stability over Time

**Figure 4:**
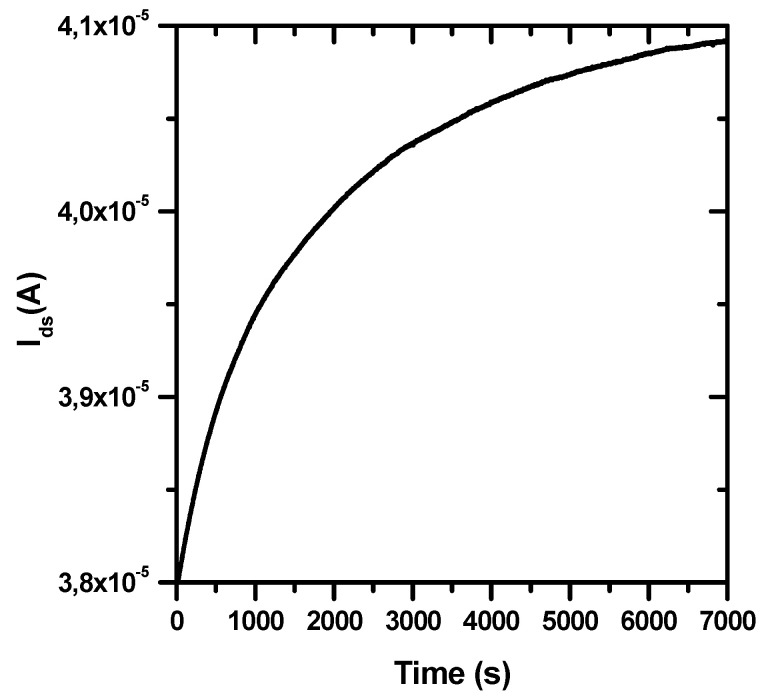
Representative behaviour of the drain-source current (I_ds_ at constant V_ds_ = 100 mV) measured by an n-type MOSFET whose gate electrode is connected with a biofunctionalized gold extended gate immersed in an electrolyte solution (PBS 100 mM solution, pH 8.0), upon the application of a constant voltage (V_ref_ = 400 mV) through a bulky Ag/AgCl reference electrode.

The starting I_ds_ current in every BioFET experiment (at constant V_ds_ = 100 mV and V_ref_ = 400 mV) is in range of 30–40 μA and it always exhibited an approximately exponential increase of a few μA before reaching a stable value, as shown in Figure 4. A similar behaviour is observed also when clean gold EG electrodes are used.

Drift of the I_ds_ in BioFET could depend on leakage due to reference electrode microfabrication [19]. Indeed, we found some instabilities (not shown) in the gate voltage when our microfabricated Ag/AgCl pseudoreference electrodes (see Figure 1) were used. Therefore, we decided to use a much more stable bulky Ag/AgCl reference electrode [34], as discussed in Section 2.2. In this case, the observed I_ds_ drift can depend only on changes of the surface potential of the gate electrode, which affects the threshold voltage V_th_ in the transfer curve [34]. An analogous drift of V_th_ has been frequently observed when oxide materials (i.e., SiO_2_, Si_3_N_4_, Al_2_O_3_, Ta_2_O_5_) are used for the BioFET gate electrode and directly immersed in the electrolyte solution [17]. This initial current drift has been described in terms of a rate-limiting ion transport from the solution to buried sites on the gate surface, and the application of a dispersive transport model led to a power-law time dependent decay of diffusivity and mobility, consistent with a stretched exponential time dependence of the gate surface charge [34].

To the best of our knowledge, no previous studies have mentioned similar current drifts when a gold EG electrode is immersed in the electrolyte solution. Nevertheless, we can speculate that, in this case, the application of V_ref_ could cause a charge translocation from the gold EG to the gate electrode of the FET, where dispersive transport may occur due to trapped states in the dielectric layer.

### 3.3. Response to p53_wt_: Extended-Gate Surface Potential Changes and Detection Limit

**Figure 5:**
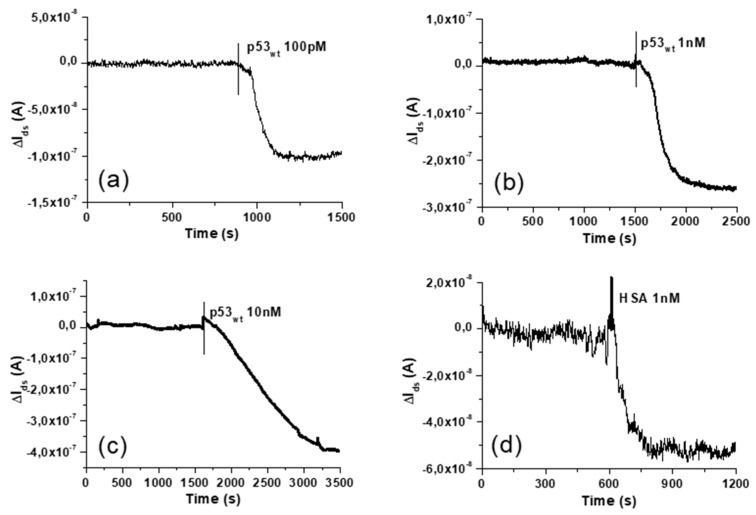
Real time acquisitions of the n-type MOSFET drain-source current I_ds_ (subtracted by the starting value) in different biosensing experiments, performed in the same conditions (V_ds_ = 100 mV, V_ref_ = 400 mV, working buffer: PBS 100 mM, pH 8.0, starting volume: 350 μL). (**a**–**c**) Single injections of 20 μL of the working buffer with diluted p53_wt_, resulting in a final concentration in the fluid cell of 100 pM, 1 nM and 10 nM. (**d**) Single injection of 20 μL of the working buffer with diluted HSA (1 nM final concentration). Vertical bars indicate the protein injection time.

After current stabilization, the sensor response to p53_wt_ has been tested by continuously recording I_ds_ while injecting small volumes (20 μL) of p53_wt_ solutions in the fluid cell. As shown in Figure 5a–c, p53_wt_ solution injection induces a decrease of I_ds_, as due to a shift of the V_th_ towards positive values, as expected since p53_wt_ bears a net negative charge at pH 8.0 [15]. Protein injections have been performed also by sequentially injecting increasing concentrations of p53_wt_. In this last case, at each step, the total I_ds_ response (I_ds_ shift with respect to the starting value) has been associated to the total p53_wt_ concentration (as obtained by cumulating the different injections). Comparable current jumps are observed at the same p53_wt_ concentration, either in single and in multiple injection experiments. Concentrations of p53_wt_ from 50 pM to 10 nM have been tested, obtaining I_ds_ jumps ranging from about −5.0 × 10^−8^ A up to about −5.0 × 10^−7^ A.

**Figure 6:**
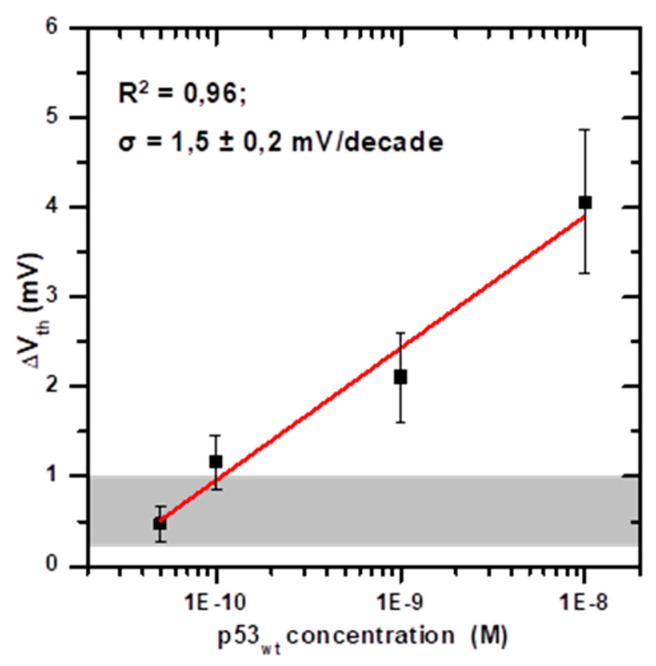
Calibration curve of the BioFET for the detection of p53_wt_, as obtained by plotting the average values of the threshold voltage (V_th_) variation as a function of the p53_wt_ concentration in PBS 100 mM pH 8.0, on a semilogarithmic scale. The error bars represent the standard deviation. The regression line (R^2^ = 0.96) is reported in red. From the slope of the regression line, the sensitivity σ = 1.5 ± 0.2 mV/decade is obtained. The grey area in the bottom represents the range of V_th_ variations obtained by injecting the working buffer with diluted other proteins (0.6 ± 0.4 mV), which can be considered as the blank signal.

The measured current jumps ΔI_ds_ have been converted in the corresponding ΔV_th_, and averaged per p53_wt_ concentration. The so-called BioFET calibration curve can thus be obtained by plotting the obtained average ΔV_th_ as a function of the p53_wt_ concentration, on a semilogarithmic scale, as shown in Figure 6 [17]. The error bars represent the data standard deviation, which likely depends on variability in the sensor microfabrication, solution preparation and volume injection, and also on the antigen concentration. The best regression line (R^2^ = 0.96), shown by the red line in Figure 6, reveals that the BioFET sensor responds to three orders of magnitude of the target concentration. Therefore, the calibration curve allows to determine an unknown p53_wt_ concentration from the corresponding measured V_th_ shift, within the above mentioned range.

From the slope of the regression line, a sensitivity (σ) of 1.5 ± 0.2 mV/decade is obtained, in agreement with the indication of the International Union of Pure and Applied Chemistry (IUPAC) [17]. Such a sensitivity value, together with the tested range of concentration, enables our BioFET as suitable tool for the assays of p53_wt_ at concentrations expected in cancer cells with p53 gene mutation (0.1 nM), as well at concentrations expected in healthy cells (1 nM), and beyond (to diagnostic diseases characterized by high level of p53_wt_ in blood). It is worth noting that the obtained sensitivity is low if compared to the limiting value of 59 mV/decade, expected if biomolecule binding would follow the Nernstian model of equilibrium potentials of ions across semipermeable membranes [17]. Such an extreme value has been previously reported for glucose [37] and urea [32] BioFETs with gold EG. However, there are significant differences between proton equilibria and macrobiomolecule binding equilibria, which imply that the Nernst model would not apply to protein binding [17]. Indeed, much lower sensitivity values (down to about 10^−3^ mV/decade) have been reported for protein biosensing via BioFET with gold EG [38].

To test the specificity of the BioFET, we have analysed the current response to the addition of two other biomolecules (BSA and human serum albumin—HSA), which also bear a negative net charge at the working buffer pH, by injecting them in the sensor fluid cell at comparable concentrations and volumes with respect to p53_wt_. A representative current response obtained as consequence of the injection of 20 μL of working buffer with 1 nM of HSA is shown in Figure 5c. The injection of HSA or BSA generally gives rise to current variations in the (–5.0 ± 3.0) × 10^−8^ A range, resulting lower than the p53_wt_ induced current changes (except for the 50 pM concentration). The corresponding V_th_ variation is in the 0.6 ± 0.4 mV range, and it is represented, in Figure 6, by the grey area at the bottom. This offset is likely due to unspecific interactions between the proteins and the EG biofunctionalized electrode [17,39], and it can be considered as a blank signal.

Determination of a blank signal allows us to calculate the BioFET LOD, which is defined by IUPAC as the smallest measure that can be reasonably detected for a given analytical procedure, and which is obtained by the blank signal incremented by a certain number of times of its standard deviation (SD), depending on the confidence level required [17]. For BioFET devices with gold EG, the LOD has been calculated by the value of the blank signal incremented by three times its standard deviation [40]. Other authors calculated the LOD as three times the standard deviation of the blank signal divided by the slope of the calibration curve [38]. We have obtained the LOD of our BioFET by calculating <V_th blank_> + k SD _blank_ per k = 1, 2, 3, obtaining LOD = 130, 190 and 290 pM, respectively. On the other hand, some authors refer to the LOD simply as to the lowest target concentration that provide a detectable sensor response visually, based on the calibration plot [28,32]. In such a case, a LOD of 100 pM is obtained for our BioFET.

A LOD of 100 pM obtained in physiological solution is a good result if compared with the previously realized BioFET for p53_wt_, for which a label-free detection of 100 nM in diluted buffer has been reported [15]. On the other hand, the LOD of our BioFET is from one to four orders of magnitude higher than those previously reported for optical biosensors (see Introduction), except for the colorimetric assay, which has a LOD of 5 nM [9]. However, our BioFET is able to detect label-free p53_wt_ in physiological buffer, while most of the previously proposed methodologies (excepted SPR [4]) are label-requiring and/or need dilution of the medium (with a few exception [3,9,11,13]). Moreover, thank to the chosen antibody, our BioFET is highly specifically sensitive only to p53_wt_, excluding the mutated forms; this avoiding misleading (unsorted) outputs. Finally, our BioFET has the clear advantages of reduced dimension, low cost and capability to work with small sample volumes, which render it a good candidate for the future development of portable devices.

### 3.4. Energetics of the Adsorption Process

The BioFET response to the target adsorption can further provide information on the energetics of the receptor-target binding, by applying models based on the Langmuir adsorption isotherm. This has been previously obtained both from real-time measurements through flow cells [41,42] and by static measurements such as in our case [26,27,39,43,44]. Within this context, and taking into account the offset observed when nonspecific adsorption occurs (ΔV_0_), the V_th_ variation can be described by:

$$\Delta V_{\mathrm{th}}=\Delta V_{0}+\Delta V_{\mathrm{th}\;\max}\times \frac{C_{p53}}{K_{D}+C_{p53}}$$

ΔV_thmax_ is the potential variation associated at the surface saturation, C_p53_ is the p53_wt_ concentration in solution, and K_D_ is the dissociation constant at the chemical equilibrium:

[p53] + [PAb1620] ↔ [p53][PAb1620]

**Figure 7:**
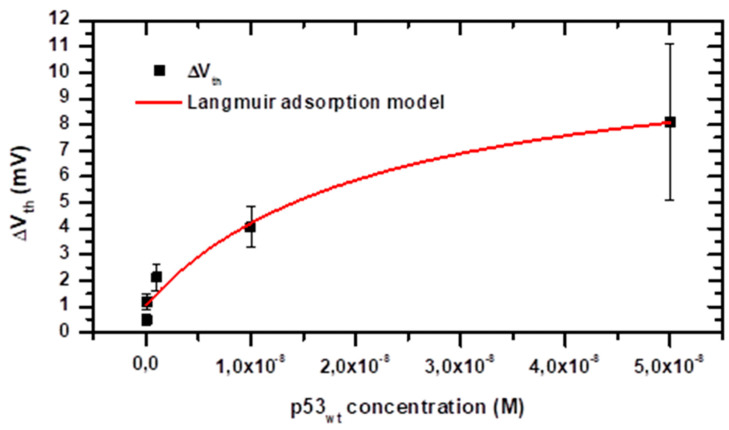
Mean threshold voltage (V_th_) changes as a function of the p53_wt_ concentration in PBS 100 mM pH 8.0 solution. The error bars represent the data standard deviation. The data have been fitted by an adapted version of the Langmuir adsorption model including an offset to take into account the blank signal (see text). The parameters extracted by fitting the experimental data according to the Langmuir adsorption model (continuous red line; R^2^ = 0.96) are: offset ΔV_0_ = 1.0 ± 0.4 mV, ΔV_thmax_ = (10.1 ± 2.2) mV, K_D_ = (2.2 ± 1.3) × 10^−8^ M.

The experimental ΔV_th_ as a function of C_p53_ have been replotted, up to the saturation level (i.e., up to 50 nM), in linear scale, in Figure 7. As for Figure 6, the experimental errors could mainly originate from variability in the sensor microfabrication, solution preparation, volume injection and antigen concentration. In the measurements performed at 50 nM concentration, in addition to the variability induced by the above mentioned issues, it should be taken into account the variability in the sensor surface preparation, which become critical in the saturation regime, when the number of available binding site on the electrode represents an upper limit to charge accumulation at the gate surface. The data have been fitted with the proposed model (red line), obtaining a good match (R^2^ = 0.96). From the fitting analysis, an offset value of 1.0 ± 0.4 mV is obtained, which is consistent with the blank signal represented by the grey area in Figure 6. The dissociation constant of the interaction among p53_wt_ and its conformational antibody PAb1620 is also obtained, as K_D_ = (2.2 ± 1.3) × 10^−8^ M. This corresponds to an affinity constant K_A_ = 1/K_D_ ≈ 4.5 × 10^7^ M^−1^ that is only slightly lower than those previously reported between p53_wt_ protein and PAb241, PAb246 antibodies or consensus ds-DNA (6.9 × 10^8^ M^−1^, 3.7 × 10^8^ M^−1^ and 1.1 × 10^8^ M^−1^, respectively [4]). Indeed, the monoclonal antibody we used (PAb1620) binds an epitope on the surface of correctly folded p53_wt_, and, upon binding, allosterically inhibits p53_wt_ binding to DNA. However, it is highly sensitive to protein unfolding or mutation (even single aminoacidic mutation) and p53_wt_ is known to assume different conformations, not all of which are recognized by PAb1620 [45]. This could partially justify the observed K_D,_ which, on the other hand, has not been so far reported in the literature. An even lower affinity (K_D_ = 3.5 × 10^−6^ M) has been reported by SPR for the binding of a peptide cloning the p53_wt_ binding site to PAb1620 [45].

## 4. Conclusions

We have developed a BioFET for the detection, in physiological-like solution, of the wild-type form of the tumour suppressor p53, whose concentration in cells and blood has a clinical significance for early diagnosis of some types of cancer and other importantly related diseases. We have implemented the device with a sensing electrode constituted by a carefully microfabricated high-purity gold electrode acting as a disposable extended gate. The sensing electrode is, on one of its ends, placed in the biological solution and, on the other end, connected to the MOSFET gate. Electrostatic (Debye) screening by the solution ions is mitigated by creating at the EG surface a Donnan-like membrane, permeable to the target biomolecules, by means of suitable PEG polymers entering in the biofunctionalization procedure. We have thus obtained a BioFET with disposable biosensing element, endowed with a quite good sensitivity (1.5 ± 0.2 mV/decade). The BioFET LOD can be as low as 100 pM with a very good specificity toward p53_wt_. The range of significance is spanning over three orders of magnitude, falling in the range of p53_wt_ concentrations that are suitable for early diagnosis and prognosis of cancer.

All these features pave the way for a future development of a related point-of-care device, provided that it is, compactly, coupled to a suitable microelectronic system able to reveal, process and display signals at high resolution and statistically significant, with the aim to reduce dimension and cost, by remaining highly competitive with standard immunoassays.
